# Anaesthetic management and clinical outcomes of balloon pulmonary valvuloplasty in dogs with severe pulmonic stenosis: a retrospective study (2024–2025)

**DOI:** 10.3389/fvets.2026.1757690

**Published:** 2026-06-18

**Authors:** Daniele Midori Kakimoto Higa, Alessandro Rodrigues de Carvalho Martins, Denise Tabacchi Fantoni, Matheus Matioli Mantovani, Adan William de Melo Navarro, Gabrielly Moreira dos Santos de Oliveira, Renata Sayuri Akabane, Guilherme Teixeira Goldfeder, Viviane Sanchez Galeazzi, Mayara Travalini de Lima

**Affiliations:** 1 Department of Surgery and Anaesthesiology, Faculty of Veterinary Medicine, Veterinary Intensive Care Unit, Unit of Training Applied to Research and Extension (UFAPE), São Paulo, Brazil; 2 Department of Surgery, Faculty of Veterinary Medicine and Animal Science, University of São Paulo, São Paulo, Brazil; 3 Faculty of Veterinary Medicine and Animal Science, Federal University of Uberlândia, Uberlândia, Brazil; 4 Goldfeder Cardiologia Veterinária LTDA, São Paulo, Brazil

**Keywords:** anaesthetic management, anaesthetic protocols, balloon pulmonary valvuloplasty, clinical outcomes, dogs, echocardiographic pressure gradient, hemodynamic instability, perioperative complications

## Abstract

**Introduction:**

Balloon pulmonary valvuloplasty (BPV) is the most common interventional procedure for treating severe pulmonic stenosis (PS) in dogs. Due to the cardiovascular compromise associated with PS, anaesthetic management must be carefully planned to ensure patient safety.

**Objectives:**

To describe the anaesthetic protocols, perioperative complications, and clinical outcomes of dogs with severe PS undergoing BPV.

**Methods:**

A retrospective review of medical records was performed for 21 dogs with severe pulmonic stenosis (echocardiographic pressure gradient > 80 mmHg) that underwent balloon pulmonary valvuloplasty between 2024 and 2025. Anaesthetic protocols were individualized based on cardiovascular assessment. Data were analyzed descriptively, and statistical analyses were performed using IBM SPSS Statistics for Windows (version 25). A significance level of *p* < 0.05 was adopted.

**Results:**

Twenty-one dogs were included, with a median age of 18 months (range: 3–84) and median body weight of 7.75 kg (range: 2.2–26). French Bulldogs were the most common breed (33.3%). Syncope (42.9%) and fatigue (38.1%) were the most frequent clinical signs. Anaesthetic induction was predominantly performed using etomidate–midazolam–based protocols (95%), and anaesthesia was maintained with mainly isoflurane combined with intravenous adjuncts. Hypotension (MAP < 65 mmHg) was the most common intraoperative complication, occurring in 66.7% of dogs, frequently requiring vasoactive support. Cardiac arrhythmias, including ventricular arrhythmias and bradyarrhythmia’s, were also observed. Balloon pulmonary valvuloplasty resulted in a significant reduction in right ventricular pressure gradient in 90% of cases.

**Conclusion:**

Balloon pulmonary valvuloplasty effectively reduced right ventricular pressure in dogs with severe pulmonic stenosis. Anaesthetic management was heterogeneous, with frequent use of etomidate- and benzodiazepine-based induction and inhalant anaesthesia combined with intravenous adjuncts. Intraoperative hemodynamic instability and arrhythmias were common, reinforcing the need for individualized anaesthetic planning and vigilant perioperative monitoring.

## 1 Introduction

Pulmonic stenosis (PS) is a congenital cardiac malformation that results in obstruction to blood flow from the right ventricle (RV) to the pulmonary artery. There are three types of pulmonic stenosis: subvalvular, valvular, and supravalvular. The most commonly observed type is valvular pulmonic stenosis (1). Diagnosis is based on the patient’s clinical history, presenting signs, electrocardiography, thoracic radiography, and echocardiography with Doppler (2).

In many dogs, particularly during the first year of life, clinical suspicion of pulmonic stenosis (PS) arises during routine health screening or preoperative evaluation following the auscultation of a systolic murmur, typically with maximal intensity over the left heart base. A substantial proportion of affected dogs are asymptomatic at the time of detection (3). However, definitive diagnosis cannot be established based on auscultation alone and requires echocardiographic confirmation, including Doppler assessment (3, 4). Murmurs persisting beyond the expected age of spontaneous resolution of physiologic (innocent) murmurs in puppies are more suggestive of underlying structural cardiac disease (5). A comprehensive echocardiographic examination—comprising two-dimensional imaging, color flow Doppler, and spectral Doppler—is essential to confirm PS, evaluate right ventricular remodeling, identify concurrent cardiac abnormalities, and determine both the anatomical location and severity of the stenosis (6). Chronic pressure overload may lead to the development of concentric hypertrophy and right ventricular dilation, adaptations that occur due to the need to generate elevated systolic pressures to overcome the gradient imposed by the stenosis (7).

Dogs with mild to moderate pulmonic stenosis (PS) may remain asymptomatic. However, in severe cases, clinical signs such as weakness, exercise intolerance, and collapse (syncope or pre-syncope) are commonly observed, which may be associated with low cardiac output, pulmonary hypoxemia, or hypotension (1, 3, 8).

In the worst cases, dogs may progress to right-sided congestive heart failure and/or experience sudden death (9). The manifestation of these signs is related to the severity of the outflow tract obstruction; therefore, treatment decisions should be based on both the severity of clinical signs and the magnitude of the pressure gradient measured across the pulmonary valve (2).

Currently, balloon pulmonary valvuloplasty (BPV) is considered the preferred interventional treatment option for valvular pulmonic stenosis in dogs with severe disease (10). This minimally invasive procedure has been well described in the veterinary literature and is associated with haemodynamic and clinical improvement in affected patients (11).

Pulmonic stenosis (PS) is most often found as an isolated condition; however, it may also be part of a complex congenital heart disease, such as tetralogy of Fallot (12). The most commonly reported associations include subaortic stenosis, tricuspid dysplasia, atrial and ventricular septal defects, patent ductus arteriosus, persistent left cranial vena cava, and azygos continuation (2).

Anaesthetic management of dogs with severe pulmonary stenosis requires specific considerations due to right ventricular pressure overload, associated concentric hypertrophy, and consequent diastolic dysfunction (13). The primary anaesthetic goals in dogs undergoing balloon valvuloplasty for pulmonic stenosis include maintenance of adequate preload and systemic vascular resistance, preservation of sinus rhythm, and avoidance of clinically significant hypotension or bradycardia in order to support right ventricular filling, coronary perfusion, and cardiac output (14). Additionally, anaesthetic management should aim to avoid excessive increases in right ventricular systolic pressure, which may contribute to tricuspid valve insufficiency, particularly in the presence of right atrial enlargement and right ventricular dysfunction (15). Preoxygenation for at least 5 min prior to induction is recommended in order to optimize myocardial oxygen reserves (16, 17). In cases of hypotension, treatment should prioritize the use of vasoactive agents, such as norepinephrine or vasopressin, aiming to increase vascular tone and maintain perfusion pressure, with beneficial effects on venous return and preload (18). Additionally, short-acting beta-blockers such as esmolol may be considered for the management of dynamic right ventricular outflow tract obstruction following balloon dilation, rather than for routine prophylactic use, and have been described in both human and veterinary literature (15, 19, 20).

Although etomidate may promote adrenal suppression when administered as a continuous infusion, this drug is known to be recommended for induction in dogs with cardiopathy due to its relative cardiovascular stability (21, 22). However, in BPV, other agents, including propofol, ketamine, and alfaxalone, administered in combination with benzodiazepines, have also been frequently used in veterinary patients undergoing balloon pulmonary valvuloplasty (13, 23). In retrospective studies of dogs undergoing balloon valvuloplasty for pulmonic stenosis, induction was most commonly performed using propofol or ketamine combined with a benzodiazepine, followed by inhalant-based maintenance with adjunctive opioid or lidocaine infusions (13, 23). A new retrospective series (44 dogs) reported similar multimodal approaches, including frequent use of propofol, alfaxalone or etomidate with benzodiazepine co-induction, and inhalant or total intravenous maintenance techniques, with a high incidence of intraoperative complications such as hypotension and arrhythmias (13, 23). More recently, dogs enrolled in a prospective study evaluating prophylactic lidocaine infusion during BPV were uniformly induced with alfaxalone and diazepam; however, lidocaine administration did not result in a significant reduction in the frequency or severity of ventricular arrhythmias (24).

Adjunctive strategies, including the use of *α*_2_-agonists such as dexmedetomidine, have been described in select cases, although their effects on heart rate and vascular resistance warrant careful consideration in patients with right ventricular outflow tract obstruction (25). Similar principles are emphasized in human pediatric and neonatal anaesthesia for critical pulmonic stenosis, where opioid-based balanced techniques aimed at minimizing haemodynamic fluctuations and preserving ventricular function have been reported (26).

Despite the importance of anaesthetic management during balloon pulmonary valvuloplasty, limited data describe anaesthetic protocols and perioperative complications in dogs undergoing these procedures. Therefore, the objective of this study was to describe anaesthetic management and immediate perioperative events in dogs undergoing transvascular cardiac interventions at a single institution. Clinical outcomes are defined as intraoperative and early postoperative anaesthetic events, including hemodynamic instability, hypoxemia, and perioperative mortality.

Given the retrospective and descriptive nature of the study, no formal hypothesis was tested.

## 2 Materials and methods

This manuscript describes a single-center, retrospective, clinical study conducted through the review of medical records from 21 dogs diagnosed with severe pulmonic stenosis (PS), as defined by a transvalvular systolic pressure gradient > 80 mmHg (1). All included patients underwent balloon pulmonary valvuloplasty between January 2024 and March 2025 at UFAPE Veterinary Hospital – São Paulo.

A total of 12 cases were excluded from the initial dataset due to missing anaesthetic records (*n* = 5), incomplete preoperative examinations and/or echocardiographic data (*n* = 2), a different cardiac diagnosis—patent ductus arteriosus (PDA) (*n* = 2), or procedures performed by a different interventional cardiologist (*n* = 3).

Preoperative data collected included age, breed, sex, body weight (kg), clinical signs (exercise intolerance, syncope, dyspnea, cyanosis, tachypnea, fatigue, collapse, seizure, and signs of right-sided congestive heart failure, including ascites), ongoing medications, hematological exams (complete blood count and leukogram), biochemical profile (urea, creatinine, albumin, alanine aminotransferase [ALT], alkaline phosphatase, and/or total bilirubin), coagulation profile (prothrombin time [PT], activated partial thromboplastin time [aPTT], and fibrinogen), echocardiography, and electrocardiography (ECG).

Intraoperative monitoring data were collected retrospectively from anaesthetic records. Recorded variables included heart rate and rhythm assessed by lead II electrocardiography, invasive arterial pressure obtained via cannulation of the metatarsal artery or, when applicable, non-invasive arterial pressure measured using ultrasonic Doppler with an appropriately sized cuff (30–40% of thoracic limb circumference). End-tidal carbon dioxide (EtCO_2_) and inspired and expired isoflurane concentrations (InsIso and ETiso) were documented using a gas analyzer and multiparametric monitor (Dräger Vista 120, Drägerwerk AG & Co. KGaA, Lübeck, Germany). Ventilatory mode, settings, and the use of positive end-expiratory pressure (PEEP) were also recorded, as well as adjustments made based on arterial blood gas analysis to optimize gas exchange during the procedure.

Anaesthetic induction and maintenance data were extracted from anaesthetic charts. Recorded variables included pre-oxygenation practices, induction agents, and maintenance of anaesthesia with volatile or injectable agents. Detailed anaesthetic protocols and drug dosages are presented in Table 1. Definitions for perioperative events were predefined for data analysis, with bradycardia defined as a heart rate < 45 beats per minute and hypotension defined as mean arterial pressure < 65 mmHg. The use of neuromuscular blocking agents, reversal drugs, and adjunct medications during emergence was also recorded retrospectively from anaesthetic records. Neuromuscular blockade was monitored using train-of-four (TOF) stimulation (Stimpod NMS450, Xavant Technology, Pretoria, South Africa), allowing titration of rocuronium and confirmation of neuromuscular recovery before reversal and extubation.

**Table 1:** Anaesthetic protocol in 21 dogs undergoing balloon catheter pulmonary valvuloplasty.

Therapy	Number of dogs (%)	Doses
Pre-anaesthetic medication		
Methadone	7 (33.3%)	0.2 mg.kg^−1^
Dexmedetomidina + Methadone	3 (14.3%)	1–2 μg.kg^−1^; 0.2 mg.kg^−1^
Butorphanol	3 (14.3%)	0.2–0.3 mg.kg^−1^
Not performed	3 (14.3%)	-
Acepromazine + Methadone	2 (9.5%)	0.01 mg.kg^−1^; 0.1 mg.kg^−1^
Acepromazine	1 (4.8%)	0.01 mg.kg^−1^
Acepromazine + Butorphanol	1 (4.8%)	0.01 mg.kg^−1^; 0.3 mg.kg^−1^
Morphine	1 (4.8%)	0.3 mg.kg^−1^
Induction protocol		
Etomidate + Midazolam + Lidocaine	12 (57.1%)	1–3 mg.kg^−1^; 0.3–0.5 mg.kg^−1^; 2–3 mg.kg^−1^
Etomidate + Midazolam + Fentanyl + Lidocaine	3 (14.3%)	2 mg.kg^−1^; 0.3 mg.kg^−1^; 2.5 μg.kg^−1^; 2 mg.kg^−1^
Etomidate + Midazolam	3 (14.3%)	1–2 mg.kg^−1^; 2 mg.kg^−1^
Etomidate + Midazolam + Fentanyl	1 (4.8%)	2 mg.kg^−1^; 0.3 mg.kg^−1^; 2.5 μg.kg^−1^
Midazolam + Lidocaine + Propofol	1 (4.8%)	0.3 mg.kg^−1^; 2 mg.kg^−1^; 5 mg.kg^−1^
Etomidate + Propofol	1 (4.8%)	2 mg.kg^−1^; 5 mg.kg^−1^
Maintenance		
Isoflurane + Lidocaine	10 (47.6%)	0.6–1.5%; 50 μg.kg.minute^−1^
Isoflurane + Lidocaine + Fentanyl	8 (38.1%)	0.6–1.5%; 50 μg.kg.minute^−1^ + 2.5–5 μg.kg.hour^−1^
Isoflurane + Lidocaine + Rocuronium	1 (4.8%)	0.6–1.5%; 50 μg.kg.minute^−1^ + 0.6 mg.kg.hour^−1^
Propofol + Lidocaine + Fentanyl	1 (4.8%)	0.2–0.3 mg.kg.minute^−1^; 50 μg.kg.minute^−1^ + 2.5–5 μg.kg.hour^−1^
Propofol + Lidocaine + Remifentanyl	1 (4.8%)	0.2–0.3 mg.kg.minute^−1^; 50 μg.kg.minute^−1^ + 0.2–0.3 μg.kg.minute^−1^

Continuous variables were expressed as mean ± standard deviation for normally distributed data and as median and range for non-normally distributed data. Data distribution was assessed using the Shapiro–Wilk test and visual inspection of histograms. Categorical variables were presented as absolute numbers and relative frequencies (percentages) and were analyzed using the chi-square test or Fisher’s exact test, as appropriate. Effect size was assessed using Cohen’s *d* to quantify the magnitude of differences between right ventricular pressures measured before and after balloon pulmonary valvuloplasty. This approach was used to complement statistical significance analysis by providing an estimate of the practical relevance of the observed reduction in right ventricular pressure. Cohen’s *d* values were interpreted according to conventional criteria, with thresholds of 0.2, 0.5, and 0.8 indicating small, moderate, and large effects, respectively (27). A significance level of *p* < 0.05 was adopted for all statistical analyses, which were performed using IBM SPSS Statistics for Windows, version 25 (IBM Corp., Armonk, NY, USA).

## 3 Results

Twenty-one dogs were included, comprising 11 females (52.4%) and 10 males (47.6%), with ages ranging from 3 to 84 months (Table 2). The most prevalent breeds were French Bulldog (*n* = 7; 33.3%), German Spitz (*n* = 3; 14.3%), and American Bully (*n* = 3; 14.3%). Shih Tzu, Cavalier King Charles Spaniel, and mixed-breed dogs were represented by two individuals each (9.5%), while Yorkshire Terrier and Schnauzer each accounted for 4.8% of the sample (Table 2).

**Table 2:** Descriptive statistics of 21 dogs undergoing balloon catheter valvuloplasty were included in the study.

Dog	Age (months)	Weight (kg)	Gender	Breed	Clinical signs	Continuous use medication	ECHO gradient (mmHg) pre valvuloplasty	RVP pre valvuloplasty (mmHg)	RVP post-valvoplasty (mmHg)
1	11	24.3	F	Mixed breed	Fatigue/syncope	0	181	103	74
2	12	2.2	F	German Spitz	Fatigue/syncope	0	137.64	70	79
3	10	11.3	F	French Bulldog	Fatigue	0	200	150	97
4	12	19	M	American Bully	Dyspnea/cough/fatigue	0	130.57	–	–
5	9	9.7	M	American Bully	Ascites	0	137.29	74	60
6	60	4.8	M	Shih-Tzu	Syncope	Discontinued^1^	132.62	141	49
7	4	6.2	M	French Bulldog	Dyspnea/syncope/cyanosis/mucosal congestion/ascites	Atenolol	141.11	95	87
8	11	5.7	F	Schnauzer	.	0	150	100	42
9	24	3.3	M	German Spitz	Syncope/cyanosis	Atenolol	157.99	128	42
10	24	10	M	French Bulldog	Asymptomatic	Atenolol	84	125	43
11	4	4.6	F	Cavalier King Charles Spaniel	Asymptomatic	0	84	79	28
12	24	10	F	French Bulldog	Fatigue	Atenolol	125.4	144	81
13	3	3.6	F	Cavalier King Charles Spaniel	Fatigue	0	125	136	50
14	8	8.5	F	French Bulldog	Cyanosis/dyspnea/fatigue	Atenolol	187	154	112
15	84	10	F	French Bulldog	Syncope	Discontinued^2^	86.43	36	56
16	48	2.2	M	Yorkshire Terrier	Convulsive syncope	0	200	81	97
17	24	5.5	M	Shih-Tzu	Fatigue/syncope/cyanosis	0	147.69	82	29^3^
18	84	7	M	German Spitz	Syncope	Atenolol	95.91	135	146
19	36	10	F	Mixed breed	Asymptomatic	0	172.06	172	120^3^
20	12	11.6	M	French Bulldog	Asymptomatic	Atenolol	125	–	54
21	36	26	F	American Bully	Asymptomatic	0	125	71	47

1 Enalapril and pimobendan were discontinued following the diagnosis of PS.

2 Furosemide and phenobarbital were discontinued after consultation with the cardiologist.

3 Dynamic obstruction after valvuloplasty.

RVP: right ventricular pressure.

Regarding clinical signs, syncope was the most frequent, observed in 9 dogs (42.9%), followed by fatigue (*n* = 8; 38.1%), cyanosis (*n* = 4; 19.0%), and dyspnea (*n* = 3; 14.3%). Two dogs (9.5%) presented ascites associated with right-sided congestive heart failure. Isolated cases of mucosal congestion (*n* = 1; 4.8%) and seizure preceding syncope (*n* = 1; 4.8%) were also reported. Five animals (23.8%) were considered asymptomatic during the preoperative evaluation.

Regarding pharmacological treatment, seven dogs (33.3%) were on continuous atenolol therapy, while 12 dogs (57.1%) had not received any medication before the procedure. Medication was discontinued in two dogs (9.5%) based on the cardiologist’s recommendation prior to the procedure, and preoperative treatment could not be confirmed in one dog (4.8%) (Table 2).

Regarding pre-anaesthetic medication, methadone was the most frequently used drug, administered alone or in combination in 12 dogs (57.1%). In seven dogs (33.3%), it was used alone at a dose of 0.2 mg·kg^−1^. Combined protocols included dexmedetomidine associated with methadone in three dogs (14.3%), using dexmedetomidine at 1–2 μg·kg^−1^ and methadone at 0.2 mg·kg^−1^. Butorphanol was used as the sole agent in three dogs (14.3%) at doses ranging from 0.2 to 0.3 mg·kg^−1^. Premedication was not performed in three dogs (14.3%). Acepromazine-based protocols were less common and included acepromazine combined with methadone in two dogs (9.5%), acepromazine alone in one dog (4.8%), and acepromazine combined with butorphanol in one dog (4.8%). Morphine was used as a single premedication agent in one dog (4.8%) at a dose of 0.3 mg·kg^−1^ (Table 1). All pre-anaesthetic medications were administered intramuscularly 15–20 min before induction, as recorded in the anaesthetic charts.

Following 5 min of pre-oxygenation via a facial mask with an oxygen flow rate of 5 L·min^−1^, anaesthesia was induced using combinations of lidocaine, midazolam, etomidate, fentanyl, and propofol, as detailed in Table 1. Anaesthetic induction was most commonly performed using etomidate-based protocols combined with midazolam, with or without additional adjuncts. Etomidate was used in 20 dogs (95.2%), at doses ranging from 1 to 3 mg·kg^−1^. Etomidate 2 (1-2) mg.kg^−1^ and midazolam 0.3 (0.3–0.5) mg.kg^−1^ combinations were used in 19 dogs (90.5%), with midazolam administered at doses of 0.3–0.5 mg·kg^−1^. Lidocaine was included as an induction adjunct in 15 dogs (71.4%) 2 (2-3) mg.kg^−1^, and fentanyl was used in four dogs (19.0%) at a dose of 2.5 μg·kg^−1^. Propofol was used as part of the induction protocol in two dogs (9.5%) at a dose of 5 mg·kg^−1^.

Anaesthetic maintenance was performed predominantly using inhalant anaesthesia combined with intravenous adjuncts. Isoflurane-based protocols were used in the majority of procedures (19/21; 90.5%). Among these, isoflurane combined with lidocaine CRI was the most frequent maintenance strategy (10/21; 47.6%), with isoflurane concentrations reported to refer to end-tidal isoflurane values (ETIso), which ranged from 0.6 to 1.5%. and lidocaine administered at 50 μg·kg^−1^·min^−1^. Additional lidocaine boluses (2 mg·kg^−1^) were administered in six dogs, as documented in the anaesthetic records.

Isoflurane combined with lidocaine and fentanyl CRI was used in eight procedures (8/21; 38.1%), with fentanyl administered at doses of 2.5–5 μg·kg^−1^·h^−1^. isoflurane was associated with lidocaine and rocuronium administered as a continuous rate infusion at a dose of 0.6 mg·kg^−1^·h^−1^.

Total intravenous anaesthesia protocols were less frequently employed (2/21; 9.5%). Propofol combined with lidocaine and fentanyl was used in one procedure (1/21; 4.8%), with propofol infusion rates between 0.2 and 0.3 mg·kg^−1^·min^−1^, lidocaine at 50 μg·kg^−1^·min^−1^, and fentanyl at 2.5–5 μg·kg^−1^·h^−1^. Another procedure (1/21; 4.8%) was maintained with propofol combined with lidocaine and remifentanil, with remifentanil administered at 0.2–0.3 μg·kg^−1^·min^−1^.

All dogs were mechanically ventilated during anaesthesia using the same anaesthetic machine and monitoring devices (Dräger Primus; Drägerwerk AG & Co., Lübeck, Germany), operating in either volume-controlled or pressure-controlled ventilation modes. Tidal volumes ranged from 8 to 15 mL·kg^−1^, with positive end-expiratory pressure set between 3 and 5 cmH_2_O. Ventilatory settings and oxygen inspiratory fraction (FiO2) were adjusted based on arterial blood gas results obtained during routine clinical monitoring.

Neuromuscular blockade was administered in all dogs using rocuronium (0.6 mg·kg^−1^ IV) immediately after endotracheal intubation. Reversal was performed at the end of the procedure with sugamadex (2 mg·kg^−1^ IV). Flumazenil (0.02–0.04 mg·kg^−1^ IV) was administered to antagonize residual midazolam effects and facilitate controlled recovery. Two dogs received acepromazine (0.005–0.02 mg·kg^−1^ IV) after extubation due to agitation during recovery. Median anaesthetic duration was 118 min (range: 30–318 min).

All procedures were performed at the same institution, using the same monitoring and anaesthesia equipment, over a relatively short study period.

One perioperative death was recorded (1/21; 4.8%). The death occurred in the immediate postoperative period following extubation.

Hypotension was the most common complication, occurring in 66.7% of dogs (14/21). Vasoactive support was required in the majority of hypotensive cases and included norepinephrine in 12 dogs (57.1%), administered at doses ranging from 0.05 to 2 μg·kg^−1^·min^−1^, either alone or in combination with vasopressin, which was also used in 12 dogs (57.1%) at doses of 0.1–2 mUI·kg^−1^·min^−1^. Dobutamine was administered in 2 dogs (9.5%) at doses of 2–10 μg·kg^−1^·min^−1^. In addition, a fluid challenge (10 mL·kg^−1^ over 15 min, guided by fasting time and pulse pressure variation) was applied in 4 dogs (19.0%). Ephedrine was administered in 2 dogs (9.5%) at doses of 0.1 mg^−1^.kg^−1^. Phenylephrine (0.1–8 μg·kg^−1^·min^−1^) was administered in 3 dogs (14.3%) with a persistent patent foramen ovale (PFO).

Bradycardia was observed either secondary to hypotension or following *α*_2_-agonist administration. In two dogs (9.5%), bradycardia occurred concurrently with hypotension and was treated with atropine (0.02–0.04 mg·kg^−1^) due to associated bradyarrhythmia. In contrast, bradycardia observed after dexmedetomidine administration was consistent with the expected baroreceptor-mediated reflex response to α_2_-induced peripheral vasoconstriction and was not consistently associated with hypotension. However, two dogs receiving dexmedetomidine developed clinically significant bradyarrhythmias requiring reversal with atipamezole.

Ventricular arrhythmias, predominantly premature ventricular contractions (VPCs), were also noted, frequently during intracardiac catheter manipulation. These episodes were managed with lidocaine boluses (2 mg·kg^−1^). Although not all dogs had a pre-existing right bundle branch block (RBBB), all dogs developed this conduction abnormality during and/or after balloon dilation.

Maropitant (1 mg·kg^−1^) was administered intravenously to six dogs for clinical signs compatible with nausea, such as hypersalivation, restlessness, and retching. All patients received intravenous metamizole (25 mg·kg^−1^) and intravenous meloxicam (0.1 mg·kg^−1^) as part of the analgesic protocol at the end of the procedure. Intravenous cefalotin (30 mg·kg^−1^) or intravenous ampicillin–sulbactam (25–30 mg·kg^−1^) was administered 30 min before the procedure.

All dogs were hospitalized for a minimum of 24 h for immediate postoperative monitoring due to the risk of complications, especially bleeding, common in cardiac interventions and potentially exacerbated by vascular manipulation and anticoagulant use.

Three French Bulldogs underwent pulmonary stent placement during the same procedure. All dogs showed significant clinical signs prior to the procedure, including cyanosis, cough, fatigue, and syncope, and were already receiving atenolol therapy.

Two of these procedures were reinterventions; in both cases, the initial balloon pulmonary valvuloplasty had been performed prior to the study period and was not included in the present analysis.

In one dog, an increased pressure gradient was documented 4 months after the initial valvuloplasty (from 96 to 125 mmHg), while the other underwent reintervention 2 years after the initial procedure due to recurrence of exercise-induced fatigue. All dogs received heparin (50–100 IU·kg^−1^ IV) before stent deployment to reduce thrombotic risk.

Bubble contrast testing identified a patent foramen ovale (PFO) in three dogs (one Cavalier King Charles Spaniel, one French Bulldog, and one Shih Tzu). In these patients, phenylephrine or vasopressin was preferentially used as haemodynamic support during anaesthesia.

In one Cavalier King Charles Spaniel, bubble contrast testing was not performed preoperatively, and PFO was not identified before the procedure. During anaesthesia, the dog developed marked hypoxemia, with a sudden decrease in oxygen saturation (SpO_2_ 68%). A transient improvement was observed after phenylephrine administration; however, the dog died after extubation in the postoperative period. The intraoperative hypoxemia was considered suggestive of a right-to-left intracardiac shunt.

The Shih Tzu exhibited evidence of right-to-left shunting associated with preoperative cyanosis and had a hematocrit of 62%, compatible with secondary erythrocytosis due to chronic hypoxemia.

At the beginning of the procedure, peripheral oxygen saturation (SpO_2_) was 92–93% with FiO_2_ 100%, and the PaO_2_/FiO_2_ ratio was 69 mmHg. Immediately before balloon inflation, SpO_2_ decreased to 85% despite phenylephrine administration (0.1 μg·kg^−1^ at 10 min).

After balloon pulmonary valvuloplasty, dynamic right ventricular outflow tract obstruction developed, with a decrease in mean arterial pressure from 69 to 60 mmHg, SpO_2_ dropping to 75%, and an increase in right ventricular pressure from 81 to 134 mmHg, consistent with infundibular obstruction.

Treatment with esmolol (50 μg·kg^−1^) combined with a fluid challenge (10 mL·kg^−1^ over 15 min) reduced right ventricular pressure to 29 mmHg and improved the PaO_2_/FiO_2_ ratio to 167.5 mmHg. Phlebotomy was not performed in accordance with current recommendations.

The French Bulldog exhibited evidence of right-to-left shunting associated with preoperative cyanosis. After balloon pulmonary valvuloplasty, dynamic right ventricular outflow tract obstruction was confirmed by transesophageal echocardiography.

A similar therapeutic approach was applied but failed to adequately reduce right ventricular pressure, leading to initiation of oral beta-blocker therapy postoperatively and consideration for future pulmonary stent placement.

Analysis of right ventricular pressures before and after balloon pulmonary valvuloplasty demonstrated a significant reduction in most patients (Table 2). Balloon pulmonary valvuloplasty significantly reduced the right ventricular pressure gradient in 90% of cases (109.26 ± 36.66 mmHg pre-procedure vs. 70.5 ± 32.8 mmHg post-procedure; *p* < 0.001; Cohen’s *d* = 1.06), confirming the effectiveness of the procedure in relieving valvular obstruction.

## 4 Discussion

Pulmonic stenosis (PS) is a congenital heart disease with a wide spectrum of clinical presentation, ranging from asymptomatic forms to severe cases accompanied by syncope, exercise intolerance, and an increased risk of arrhythmias and cardiovascular collapse (9). In this study, the majority of patients presented clinical signs at admission, highlighting the importance of detailed cardiologic assessment prior to any anaesthetic or interventional procedure.

A proportion of dogs in the present cohort were receiving atenolol therapy prior to the procedure, reflecting common clinical practice in patients with severe pulmonic stenosis. Beta-blockers are frequently prescribed to reduce heart rate, myocardial oxygen consumption, and the risk of dynamic right ventricular outflow tract obstruction (19). A study by Gomart et al. (7) showed that dogs receiving preoperative atenolol exhibited lower intraoperative heart rates compared with untreated dogs; however, no significant differences were observed in the frequency of ventricular arrhythmias, the need for antiarrhythmic therapy, vasopressor or positive chronotropic support, fluid boluses, or procedure duration. Based on these findings, preoperative atenolol administration does not appear to confer a clear protective effect against perioperative arrhythmias or hemodynamic instability (7). Given the retrospective and descriptive nature of the present study, and the absence of a comparator group, no conclusions can be drawn regarding the influence of atenolol on anaesthetic outcomes, and its use should be interpreted as part of baseline medical management rather than a determinant of perioperative events.

Methadone was frequently included in premedication protocols, being administered in approximately half of the procedures. Methadone was administered at low doses (0.1–0.2 mg·kg^−1^), aiming primarily at anxiolysis and attenuation of sympathetic responses rather than profound analgesia. Given the minimally invasive and low-nociceptive nature of balloon pulmonary valvuloplasty, the limited analgesic contribution of low-dose opioids is unlikely to have impacted perioperative stability. Notably, the use of methadone at these doses was not associated with hypotension, or increased vasopressor requirements (*p* = 0.785), supporting its hemodynamic safety within a multimodal anaesthetic approach.

A notable finding of the present study was the high prevalence of anaesthetic induction based on the combination of etomidate and midazolam. In this cohort, episodes of hypotension were not observed immediately after induction but occurred predominantly during the interventional phase of the procedure. This temporal pattern suggests that haemodynamic instability was more likely related to procedural factors—such as balloon inflation and intracardiac manipulation—than to the induction phase itself.

From a pharmacological perspective, etomidate is recognized for its myocardial-sparing properties and preservation of sympathetic tone, whereas midazolam is associated with minimal direct myocardial depressant effects and limited haemodynamic impact (28, 29). The combined pharmacodynamic profile of these agents is therefore considered appropriate for patients with cardiovascular compromise. Although the study design does not allow causal inference between the induction protocol and the haemodynamic profile observed, the temporal pattern identified is consistent with previous veterinary and human cardiac anaesthesia studies, in which haemodynamic disturbances were primarily attributed to surgical or interventional stimuli rather than to the induction phase (30).

Importantly, anaesthetic induction was individualized according to patient-specific considerations. In dogs at risk of intracranial hypertension, such as the patient with hydrocephalus included in this study, total intravenous anaesthesia with propofol was preferentially selected due to its neuroprotective properties and its ability to minimize increases in intracranial pressure (31). This individualized approach highlights that anaesthetic choices were guided by physiological priorities rather than rigid protocol application.

Lidocaine was frequently incorporated as an intravenous adjunct during anaesthetic maintenance as part of a multimodal anaesthetic strategy, rather than for prophylactic antiarrhythmic purposes. Intravenous lidocaine has been shown to reduce inhalant anaesthetic requirements and may contribute to improved hemodynamic stability by limiting excessive anaesthetic depth (32). In this context, the use of lidocaine in the present study should be interpreted as an anaesthetic-sparing adjunct aimed at physiological stability rather than as a routine intervention to prevent ventricular arrhythmias.

Despite the availability of total intravenous anaesthesia techniques, inhalant anaesthesia—predominantly isoflurane—remained the main maintenance strategy in the present Cohort, usually combined with intravenous adjuncts and neuromuscular blockade. This approach differs from that reported by some referral centers that favor TIVA or PIVA (23); however, inhalant anaesthesia continues to be widely used in pediatric cardiac anaesthesia, including in patients with congenital heart disease, when combined with strict ventilatory control and invasive monitoring (33). Evidence from human cardiac anaesthesia suggests that volatile anaesthetics and TIVA may offer comparable outcomes when appropriately managed (34, 35). Within this context, the frequent use of isoflurane in the present study should not be interpreted as inferior, but rather as part of a multimodal anaesthetic strategy aimed at balancing depth of anaesthesia, hemodynamic control, and rapid titratability during interventional procedures (34).

The use of neuromuscular blocking agents during anaesthetic maintenance should be interpreted within the context of institutional practice patterns and available resources. In contrast to practices reported in some institutions where the routine use of neuromuscular blockade is more restricted, its earlier and more frequent adoption in the present cohort reflects a deliberate strategy aimed at optimizing surgical conditions and ventilatory control. Neuromuscular blockade was employed to ensure complete immobility, improve patient–ventilator synchrony, and enhance surgical field stability, particularly during procedures requiring precise instrumentation. Importantly, neuromuscular blockade was always associated with adequate anaesthetic depth and analgesia, mitigating concerns related to awareness or inadequate hypnosis. Neuromuscular blockade was systematically monitored using train-of-four stimulation, reinforcing that rocuronium administration was guided by objective monitoring rather than routine or empirical use.

During recovery, flumazenil was administered in selected cases to antagonize residual benzodiazepine effects; however, post-extubation agitation was not consistently alleviated, indicating that factors other than residual midazolam may have contributed to recovery behavior. In a small number of cases, acepromazine was administered after extubation to manage agitation, reflecting individualized post-anaesthetic management rather than protocol-driven intervention.

Hypotension was defined as a mean arterial pressure below 65 mmHg (36), a threshold commonly adopted in both veterinary and human anaesthesia to minimize the risk of organ hypoperfusion. In the present study, hypotensive episodes were primarily observed during the interventional phase of the procedure rather than during anaesthetic induction, reinforcing the role of procedural factors in hemodynamic instability. Importantly, hypotension was promptly identified through invasive arterial blood pressure monitoring and rapidly corrected using volume expansion and vasoactive support when indicated. No dog developed persistent hypotension or clinical signs attributable to inadequate tissue perfusion. With the exception of one postoperative death related to procedural complications, all animals recovered from anaesthesia and were discharged without evidence of anaesthesia-related morbidity.

Ventricular arrhythmias, predominantly ventricular premature complexes, were mainly associated with mechanical catheter manipulation within the heart. Although lidocaine is widely used as first-line therapy for ventricular arrhythmias, a prospective randomized trial involving dogs undergoing balloon pulmonary valvuloplasty demonstrated that prophylactic lidocaine administration did not reduce either the incidence or severity of ventricular ectopy (24). These findings suggest that routine prophylactic antiarrhythmic therapy may not be justified and should instead be reserved for selected clinical scenarios, reinforcing that immediate communication with the interventional team and prompt adjustment of catheter positioning remain the most effective strategies to reduce myocardial irritation (24).

Bubble contrast testing is essential for detecting patent foramen ovale (PFO), allowing the identification of intracardiac shunts by evidencing the passage of bubbles from the right to the left atrium before anaesthesia and guiding safer anaesthetic management (6). In the case of the Cavalier King Charles Spaniel that died postoperatively, the absence of the test contributed to the lack of prior PFO detection. The sudden desaturation observed during the procedure suggests a right-to-left shunt due to probable increased right atrial pressure, resulting in severe hypoxemia (37). The transient response to phenylephrine indicates that manipulation of systemic vascular resistance can influence shunt direction, but not always sustainably. Thus, prior identification of PFO is critical for anaesthetic planning and risk reduction. In patients at higher risk for right-to-left shunting, the use of drugs that preferentially increase left ventricular afterload, such as phenylephrine, may help maintain adequate pulmonary flow and prevent paradoxical hypoxemia (37).

The management of dynamic right ventricular outflow tract obstruction (“suicidal right ventricle”) observed in some patients followed literature recommendations (38). Esmolol (50–200 mcg·kg^−1^ IV) was used to reduce ventricular contractility and infundibular obstruction, while fluid challenge optimized Starling forces and reduced blood viscosity, contributing to more stable hemodynamics (38). Although it is not possible to definitively predict which patients will develop dynamic obstruction after valvuloplasty, prophylactic beta-blocker administration and fluid challenge immediately before the procedure may be recommended in cases of severe PS (38).

Secondary erythrocytosis detected preoperatively in the Shih-Tzu reflects a physiological adaptation to hypoxemia. Literature reports that phlebotomy in hypoxemic patients may reduce oxygen-carrying capacity and worsen tissue oxygenation and is therefore contraindicated (39). The conservative approach adopted was appropriate.

Assessment of coronary anatomy is also an important step in planning valvuloplasty in dogs with PS, as anomalous coronary courses may pose risks during balloon dilation. When echocardiographic visualization is limited, especially in brachycephalic breeds, angiography, selective coronary catheterization, or cardiac computed tomography angiography are recommended (6). No cases in this series presented coronary anomalies.

In this study, syncope was the most frequent clinical sign, followed by fatigue, cyanosis, and dyspnea, consistent with previous reports describing the prevalence of such symptoms in moderate to severe PS (40). Breed predisposition has been reported in English Bulldogs, French Bulldogs, American Staffordshire Terriers, and Boxers (2). In this study, the highest incidence occurred in French Bulldogs and American Bullies, possibly related to the increased popularity of these breeds in Brazil, consanguineous breeding, and lack of reproductive control programs, which may contribute to a higher frequency of congenital heart disease.

Several factors may explain differences between earlier reports and the overall findings of the present study. Long retrospective periods are inherently subject to temporal heterogeneity in anaesthetic protocols, monitoring standards, and perioperative decision-making. In contrast, the present cohort represents a contemporary and relatively homogeneous population, managed within a short time frame at a single institution. The routine use of lung-protective ventilation strategies, modern invasive monitoring, systematic neuromuscular blockade with rocuronium, and a standardized and experienced anaesthetic team may have contributed to improved physiological stability and reduced variability in perioperative management.

This study has several limitations that should be acknowledged. Its retrospective design and relatively small sample size may introduce selection and information biases. Although the study was conducted over a relatively short period (1 year), minor differences in ventilatory strategies or operator practices may have influenced outcomes, and not all patients received standardized ventilatory management. Consequently, caution is warranted when interpreting the results. Finally, the findings should be considered in the context of modern pediatric cardiac anaesthesia, recognizing that advances in perioperative care may differ between institutions and over time.

## 5 Conclusion

This retrospective study describes anaesthetic management and perioperative events in dogs with severe pulmonic stenosis undergoing balloon pulmonary valvuloplasty Etomidate- and benzodiazepine-based induction protocols were frequently used, and anaesthetic maintenance was predominantly performed using inhalant anaesthesia combined with intravenous adjuncts. Hemodynamic instability and arrhythmias were commonly observed during the intraoperative period, often requiring early recognition and immediate treatment of complications, highlighting the importance of vigilant monitoring and prompt recognition and management of perioperative complications.

## Data Availability

The original data supporting the conclusions of this article will be made available by the corresponding author upon reasonable request.
